# Estimating the link between service-user patient safety perceptions, incidents and subsequent contagion in acute mental health wards

**DOI:** 10.1186/s12888-024-06261-6

**Published:** 2024-11-28

**Authors:** John Baker, Sarah Kendal, Charlotte Sturley, Gemma Louch, Chris Bojke

**Affiliations:** University of Leeds, Leeds, UK

**Keywords:** Digital technology, Evaluation study, Patient involvement, Patient safety, Acute inpatient mental health, Mixed methods, Statistics, Nursing research

## Abstract

**Background:**

Safety incidents are common in adult acute inpatient mental health services, and detrimental to all. Incidents spread via social contagion within the ward, but social contagion is difficult to quantify. Better measures of social contagion could support a milieu in which safety incidents are less likely to be prolonged, spread, or repeated, with widespread benefits.

The WardSonar project, based in the United Kingdom (UK), developed and evaluated a prototype digital safety monitoring tool to collect real-time information from patients on acute adult mental health wards, about their perceptions of ward safety. A prototype Wardsonar tool was developed from a collaborative, co-design approach, and implemented in real-world hospital settings. The current study aimed to understand whether the tool can help to predict incidents, by examining (i) the feasibility of capturing real-time feedback from patients about safety and (ii) how the resulting data related to quality and safety metrics.

This study was registered as ISRCTN14470430 on 10/January/2022.

**Method:**

Patients can record real-time perceptions of ward safety using the tool, and staff can access these as anonymous, aggregated data*.* The tool was implemented in the UK in six National Health Service adult acute mental health wards. A novel approach to analysis involved construction of an hour-by-hour dataset over each ward. This revealed relationships between quantity and content of patient reports, staffing, time of day, and ward incidents, per ward.

**Results:**

There is strong evidence that an incident leads to increased probability of further incidents within the next four hours. This supports the idea of social/behavioural contagion and puts a measure on the extent to which the contagion persists. COVID-19 impacted the research processes.

**Conclusions:**

There is potential to use the WardSonar digital tool for proactive real-time safety monitoring, to identify developing incidents and help staff to facilitate timely preventative or de-escalating interventions. Further refinement and testing in a post COVID-19 context are needed.

**Trial registration:**

ISRCTN14470430 https://doi.org/10.1186/ISRCTN14470430. Registered 10/January/2022.

## Background

Patient safety incidents are defined by NHS England as “any unintended or unexpected incident which could have, or did, lead to harm for one or more patients receiving healthcare” [1]. Mental health services report high levels of safety incidents (‘incidents’) involving patients and staff [1, 2]. According to United Kingdom (UK) government records for 2020–21, 300,703 incidents were reported in mental health services in England [3]. This is a key concern of the Care Quality Commission [4] and a National Health Service (NHS) priority [5, 6]. Although concerns about patient safety on acute mental health wards are widespread, international data are limited [7].

Detailed data from acute mental health wards show that the most frequently occurring incidents involve violence and self-harm [8, 9]. Incidents on acute mental health wards have been associated with increased costs related to the increased one-to-one nursing required for the use of restraint, seclusion, rapid tranquilisation; and physical and psychological harms, which may increase the length of a patient’s hospital stay and have a negative impact on their health-related quality of life [10]. Likewise, as incidents are also detrimental to staff wellbeing, they can result in associated costs of replacement staff and staff support [11]. In addition, one incident on a mental health ward may increase the probability of further incidents occurring via behavioural contagion, whereby behaviours can spread amongst the community [12–16]. Therefore, successfully avoiding one incident may bring further positive benefits by reducing the probability of future incidents [12, 13, 15, 17].

### Safety on mental health wards

According to patient discourses, a safe inpatient mental health setting is a place where it is not dangerous to be vulnerable [18]; and patients have a sense of being recognised as a whole person [19] and some feeling of control within an uncertain world [20]. However, organisational priorities tend towards identification and risk management of violence, aggression, self-harm or suicide by patients [21–24]. Furthermore, patient safety as an academic discipline has traditionally focused on acute physical health care settings, both empirically and theoretically. It is widely acknowledged that patient safety in mental health care contexts has been a neglected area of research [25–27].

Patients’ definitions of safety [28–30] can be absent from service-oriented perspectives. Incident reporting systems consequently fail to capture the spectrum of patients’ safety concerns, e.g. around bullying, intimidation, racism, aggression, drug and alcohol use, or theft of personal property [31]. There is potential for iatrogenic harm even when procedures are correctly followed and best practice is observed [28, 32–37]. Incidents have psychological consequences for those involved directly or indirectly (e.g. as witnesses) [38], such as anger, fear and anxiety. In addition, where organisations focus on avoiding risk, ward cultures can be characterised by ineffective and potentially harmful defensive practices [30, 39].

#### Behavioural contagion

Behavioural contagion (a type of social contagion [14, 16, 40]) refers to the tendency for people to repeat a behaviour after others have performed it [41], in a process similar to the transfer of emotions between staff and patients in a healthcare setting [42]. Behavioural contagion has been identified in studies of self-harm, aggression and assaults [12, 13, 15, 43], suicide and deliberate self-harming behaviour [13, 43, 44]. Research into the dynamics of violence on adult mental health wards found that patient aggression and self-harm incidents clustered temporally, both within a specific day and also in adjacent days, indicating that behavioural contagion was a factor [12].

#### Safety research priorities

Patient involvement is a cornerstone of policy and practice [45] and a priority for mental health research [26]. Patients and staff have expressed different interpretations of safety [46], yet, despite the policy focus, health services collect very few patient-reported safety data [47]. Adverse incident reporting mechanisms in NHS hospitals may not effectively identify all such incidents [48–50], yet safety data could be enhanced by incorporating patients’ perspectives on safety [28, 29, 46, 51, 52].

Since organisational incident reporting tends towards a narrow definition of safety, staff may not be alert to psychological harms experienced by mental health inpatients as a result of staff behaviour, treatments, or other patients. Consequently, patients may have difficulties raising concerns with staff while staying on acute mental health wards.

**Fig. 1:**
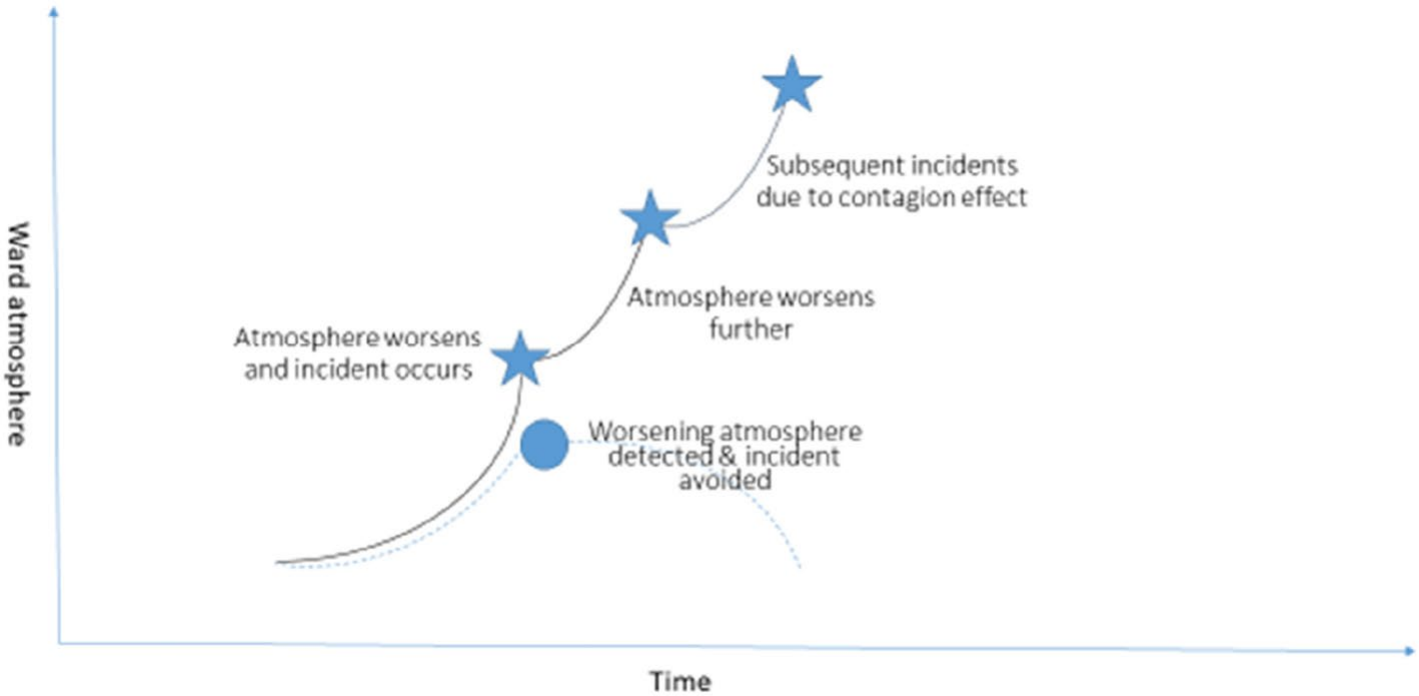
Diagrammatic overview of how a real-time intervention could prevent incidents and subsequent contagion

There are established strategies for measuring harms that have already occurred, but approaches to understanding safety of care in real-time are more limited [53]. Prospective clinical surveillance is particularly important within the acute mental health context, because of the possibility of rapid fluctuations within the interpersonal dynamics of the inpatient group and between patients, staff and the environment. Individual patient needs create immediate knock-on effects for other patients, their quality of care and their safety. Real-time data have been found to support staff to respond proactively to safety issues [54]. Therefore, the premise for the current study was that if patients were given the opportunity to report safety issues in real-time, staff could potentially respond and intervene to contain incidents and prevent incident contagion (see Fig. 1).

#### Research context

The current paper reports on the statistical evaluation of a prototype digital tool for collecting real-time information, from patients on acute adult mental health wards, about their perceptions of ward safety. The ‘WardSonar tool’ took the form of a Web app that was developed and tested within a large mixed methods project. The app was accessed via a tablet computer which staff took around the wards and encouraged the patients to complete. The patient interface consisted of three questions that used a weather analogy, e.g. ‘How does the ward atmosphere feel right now?’ and patients could select an option, such as ‘Calm’ or ‘Stormy’ and add free text if they wished.

Development and implementation processes are reported elsewhere [55, 56]. The project used a collaborative, co-design approach led by a lived experience expert on the core research team. Patients with capacity to consent were invited by staff to use the tool (i.e. the Web app) to submit their perceptions of safety, and staff could use it to access real-time anonymised and aggregated patient safety reports. Patients were free to engage or not engage with the tool.

Supporting people to ensure that they have a meaningful experience of participation is vital to health services research. The current study was developed from principles of equality, diversity and inclusion consistent with the INCLUDE guidance [57] and informed throughout by stakeholder and lay input.

The development of the WardSonar patient safety monitoring tool was a response to NHS England’s Patient Safety Strategy [6], specifically, Digital Clinical Safety Strategy [58] research priorities and National Patient Safety Strategic Research Needs [59] themes. These strategies promote digital technologies as potential solutions to patient safety challenges and encourage the development of innovative approaches to measuring and monitoring patient safety, to help understand how patient safety data collection methods can be used in real-time interventions. Digital technology could also support much-needed improvements [60] in quantitative reporting within nursing research.

### Aim and objectives

The immediate aim of the research reported in the current paper was to conduct a statistical analysis of data collected via the tool, with a view to exploring the (i) feasibility of capturing real-time feedback from patients about safety and (ii) how the resulting data related to quality and safety metrics.

### Method

**Fig. 2:**
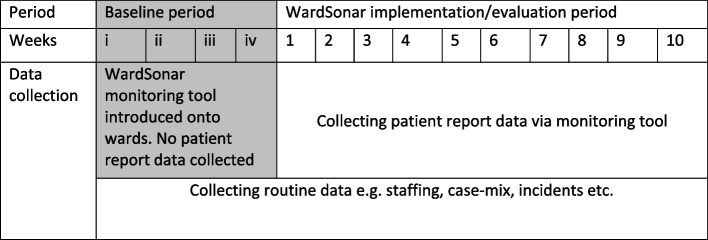
Schedule for ward-based implementation and evaluation of WardSonar monitoring tool

The tool was introduced onto three wards in each of two participating NHS Trusts in the North of England, i.e. six adult mental health acute /PICU wards (pseudonyms: Apple, Bramble, Cherry, Damson, Elderflower, and Fir). Cherry and Damson wards were Psychiatric Intensive Care Units (PICUs). A four-week baseline/pre-implementation period (Fig. 2) was followed by a 10 week implementation period on each ward (weeks i-iv and weeks 1–10 respectively in Fig. 2). Implementation and evaluation ran from January-May 2022, with staggered start dates for each of the six wards. The focus of the analysis reported here is on these six 10-week implementation periods.

#### Procedure

During the implementation period, ward nurses or healthcare support workers invited patients to report their perception of ward atmosphere via the WardSonar tool up to three times a day. The patient interface of the tool asked about the ward atmosphere (very calm to very stormy), the perceived improvement or deterioration, and factors contributing to this. These data were made available to staff in real-time via a dashboard which included user-friendly graphics.

#### Routine data collection

The two NHS Trusts provided routine incident data for the six wards for 1st January 2022 to 31st May 2022. The data contained information on the date the incident occurred, the date the incident was reported, the location where the incident occurred, the type of incident and level of harm caused by the incident.

#### Real-time analyses: data sources

##### WardSonar monitoring tool data

Statistical analysis involved construction of an hour by hour dataset over each ward. This was a fairly novel approach as previous research has tended to analyse by shift level, which may lack the granularity to pin down the idea of contagion. The number of WardSonar submissions (i.e. patient reports) per ward was compiled for each hour of each day across the 10-week implementation period. Ward atmosphere and direction scores were recorded for each hour at which there was a completed WardSonar submission. Where there were multiple completed submissions during a one-hour time period, the median average score was computed.

##### Incident data

This consisted of a total of 1,522 incidents reported across the six wards between 1st January and 31st May 2022. For the two PICU wards (Cherry and Damson), incidents that occurred in seclusion areas were combined with those on the main ward for analysis.

Date, location, category and level of harm were recorded for all incidents, but time of incident was missing in 301 cases (20% of sample). The proportion of records missing time varied by ward. Multiple imputation techniques were used to assign incidents with missing time to specific 1-h time slots, for inclusion in the statistical analysis. Harm was measured as a dichotomous variable where 0 = no harm and 1 = harm, derived from the incident harm measures used by the two Trusts.

##### Staffing data

This consisted of the number of staff on each ward by hour, which was calculated based on the shift times for each shift type, as provided by the NHS Trusts, and the number of staff on each shift by date.

##### Case mix data

This consisted of counts of patients by date and ward for each day during the 10-week implementation period and were compiled by ward.

#### Data matrix

A matrix of counts by ward of patient, staff, incidents and WardSonar submissions per hour for each day in the 10-week implementation period was compiled. Additional variables were derived. For the three wards with available case mix data, the staff-to-patient ratio was calculated at each hour and compared to the mean average staff/patient ratio for the time period.

A nominal WardSonar atmosphere response variable (positive atmosphere, negative atmosphere, medium atmosphere, no response), a WardSonar atmosphere direction response variable (getting better, getting worse, the same, no response), a volume of responses count and a recording of the ‘worst’ atmosphere response were created and used in a regression analysis. The hours were allocated to four time periods: morning 08:00–10:59; midday 12:00–14:59; afternoon 15:00–19:59; and evening 20:00–22:00 to account for any systematic variation across time.

The data were organised by hour. The night-time period (22:00–08:00) was excluded from the statistical analysis as there were very few WardSonar submissions during this period and the relationship between explanatory variables and dependent variable is expected to differ across night and day periods.

#### Statistical modelling

Several statistical models were used to explore the relationships between wards, WardSonar use and occurrence of incidents over time. Due to differing types of outcome measures, different types of models were used, ranging from zero-inflated negative binomial models for count data with a high proportion of zero counts, to ordinal logistic regression for ordered response data. However, the underlying structure of the explanatory variables, the design matrix, was consistent across all models. It comprised: ward-level fixed effects to capture time-invariant ward-specific effects; time of day variables and lagged values of incidences of WardSonar responses. A simple linear trend was also included to determine any systematic deviation in use or response over time. Patient/Staff ratios were not included in the analysis as the data were not considered universally valid.

#### Ethical approval

Ethical approval was obtained in November 2021 (detail provided below).

## Results

### Descriptive summary of WardSonar submissions, incidents and staffing data

There were 627 WardSonar submissions (602 completed submissions and 25 abandoned submissions) across the six wards over the 10-week implementation period. Table 1 shows average hourly volumes and case-mix for staffing and patients; the total observed incidents; the number of WardSonar submissions; and average hourly WardSonar scores by ward, split by day and night-time periods.

**Table 1:** Summary of WardSonar patient submissions, incidents and staffing levels by ward over the 10-week WardSonar implementation period

Ward	Time of day	Average staff	Average registered staff	Average unregistered staff	Average patients	Average patient-staff ratio	Incidents	Incidents Harm	Completed Ward Sonar submissions	Abandoned Ward Sonar submissions	Average ward atmosphere	Average ward direction
Apple	Day	7.3	2.6	4.7	22.5	3.5	119	42	66	2	Calm	The same
Apple	Night	6.2	2.0	4.3	22.5	3.9	37	14	1	1	NA	NA
Bramble	Day	8.0	2.3	5.7	NA	NA	58	18	41	1	Calm	The same
Bramble	Night	8.2	2.0	6.3	NA	NA	12	2	4	0	NA	NA
Cherry	Day	9.4	2.7	6.6	11.7	1.4	31	9	211	8	Calm	The same
Cherry	Night	7.1	1.5	5.6	11.7	1.8	2	0	5	0	NA	NA
Damson	Day	10.8	2.9	7.9	NA	NA	79	19	105	6	Calm	The same
Damson	Night	11.9	2.2	9.7	NA	NA	13	2	0	1	NA	NA
Elderflower	Day	7.2	2.4	4.8	21.3	3.4	48	6	58	2	Calm	The same
Elderflower	Night	6.5	2.0	4.5	21.3	3.7	18	3	0	0	NA	NA
Fir	Day	8.7	2.5	6.3	NA	NA	71	29	108	4	Calm	The same
Fir	Night	8.6	1.7	6.9	NA	NA	10	5	3	0	NA	NA

*NA* not applicable

#### Data entry patterns

There were some instances of multiple submissions at exactly the same date and time. As data entry patterns may reflect ward routines (e.g. group meetings, shift changes etc.) it was possible that a group of patients could be entering data during the same short period of time (potentially multiple entries inputted within one minute). There also appeared to be some repeat submissions (with the same comments), but as they were indistinguishable from duplicates or different patients entering the same information, these records were retained within the analysis data set. The number of completed WardSonar submissions varied by ward. On average, there was no perceived change in ward atmosphere on any of the six wards.

#### Incident data summary

In total, 624 incidents were reported during the implementation period. Of these, 498 cases contained the time of the incident. Apple ward had the highest number of (complete case) incidents (*n* = 156) and Cherry ward had the fewest (*n* = 33). 149 (30%) of the incidents with complete case information were classed as causing harm. The proportion varied by ward, with 42% (*n* = 34) of incidents on Fir ward classed as causing harm, compared to 14% (*n* = 9) of incidents on Elderflower ward.

##### Patient/staff ratios

Average patient/staff ratios were slightly higher during night-time hours than during daytime hours on Apple, Cherry and Elderflower wards. On all six wards the average number of registered staff was higher during daytime hours compared with night-time hours; but on Bramble, Damson and Fir wards the average number of unregistered staff was higher during night-time hours than daytime hours.

##### Day/night incident patterns

There were very few, if any, submissions during night-time hours on any of the six wards. There is some variation by ward in the pattern of submissions by time of day. The pattern of submissions on by day of week also varied by ward.

As with submissions, on all six wards there were fewer incidents during night-time hours compared with daytime hours. There was, however, some variation by ward in the pattern of incidents by time of day.

##### Ward atmosphere

When the ward atmosphere was rated as calm (“Calm” or “Very Calm” categories combined), the most common reason given for this on all but one ward was “The staff”. Similarly, the most common reason given for the ward direction “Getting calm” on all but one ward, was “The staff”. In contrast, when the ward atmosphere was rated as Stormy (“Stormy” or “Very stormy” categories combined), the most common reason on all but one ward was “The other patients”.

**Fig. 3:**
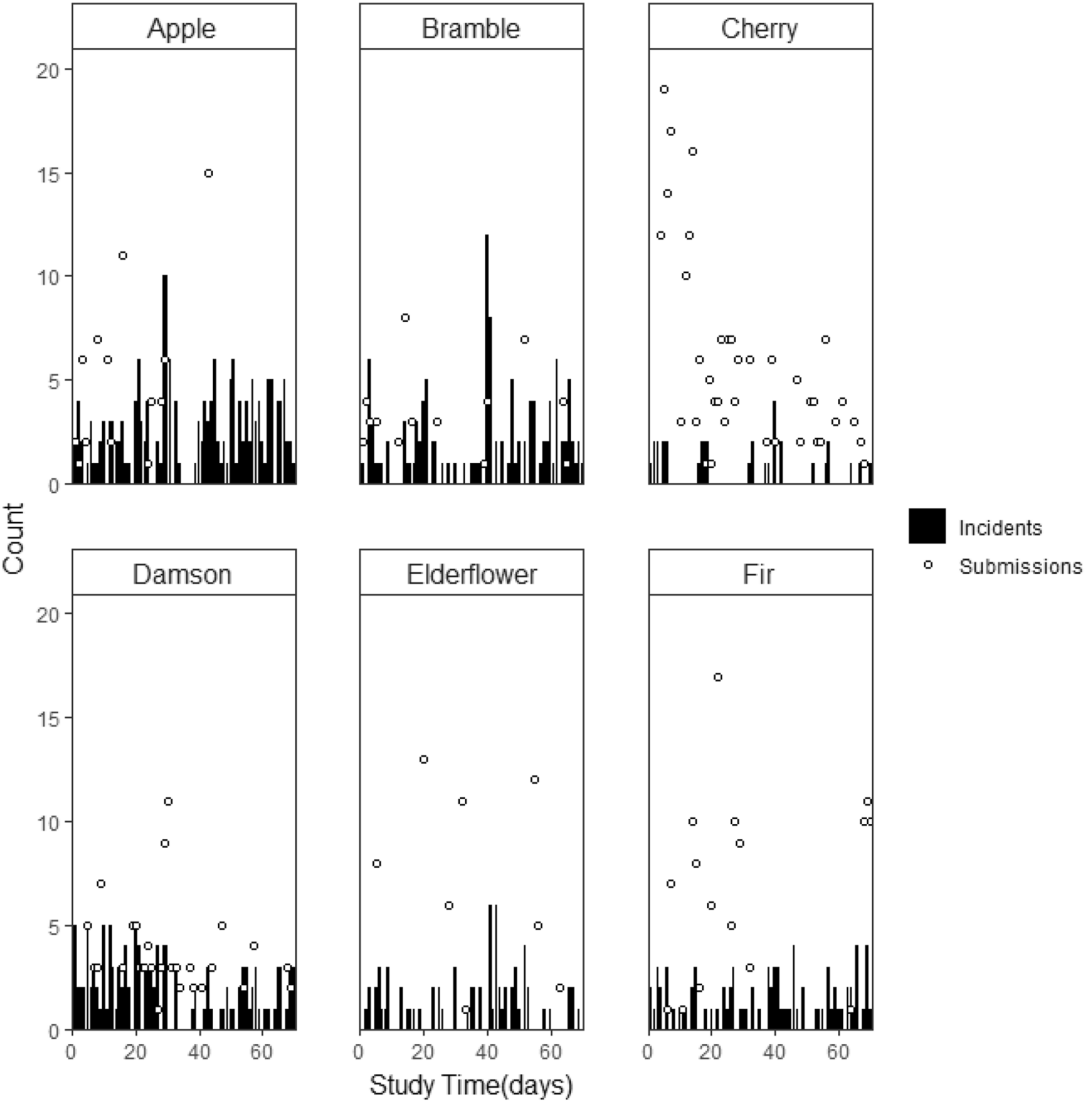
WardSonar device submissions and incidents by ward over 10-week WardSonar implementation period. A dot represents a day with 1 or more submissions

Figure 3 shows WardSonar submissions and incidents by ward over the 10-week WardSonar implementation period. Cherry ward had the most WardSonar submissions but the number of submissions tailed off over the implementation period. Submissions on Apple ward increased over the first couple of weeks of the implementation period, dropped off and then increased again in week 7, after which no more submissions were recorded. Submissions did not appear to follow any clear pattern on the other wards and there were gaps in the collected data, during which no WardSonar submissions were recorded. In particular, there were large periods with no submissions on Fir and on Elderflower ward. Where there were peaks in incidents, there appeared to be few (if any) WardSonar submissions.

### Exploratory regression analysis

Three exploratory regression analyses were used to identify any systematic patterns between wards, time, incidents, submissions and types of submission:

- Count of submissions as a function of ward, time and lagged incidents
- Type of ward atmosphere submission as a function of ward, time and lagged incidents
- Probability of an incident occurring as a function of ward, time, lagged incidents and lagged WardSonar responses (type/volume).

Of interest was whether it was feasible to measure intertemporal atmosphere contagion as a result of previous incident and whether information obtained from tool submissions may be used to predict the likelihood of incidents, above and beyond alternative information, such as knowledge of recent previous incidents.

All models were estimated using SAS 9.4 [61] analytical software. All models had a similar design matrix and consisted of: fixed effect / dummy variable for each ward to capture time-invariant underlying tendencies; a dummy variable for time of day (morning 8am – 10:59am; midday 12:00 −14:59; afternoon 15:00–19:59 and evening 20:00–23:00); a weekend dummy variable to capture any systematic impact of weekends; and a series of lagged variables indicating whether there had been an incident occurring in each of the previous 5 h. The rationale for the lagged incidents variables was to determine whether there was any evidence of social or behavioural contagion. Patient/staff ratios were not measured systematically across wards and so were excluded from the analysis. Finally, a days since trial started (days elapsed) was included as a measure of the impact of time on the study. Modifications, such as the type of lagged variables, were made to the design matrix to accommodate additional nuances of each analysis.

#### Volume of submissions

Graphical inspection of the volume of submissions revealed differing patterns across wards, time of day and duration of study; alongside a mix of other notable factors such as a lack of submissions during weekend for Fir ward and long periods of non-response within the implementation period (e.g. Apple ward after 44 days, Bramble between 25 and 38 days, Elderflower between 34 and 54 days and Fir between 33 and 63 days). Submissions also tended to be made in clusters when any submission was made. Given the large number of periods where no submissions were made, combined with a clustering of submissions, conventional ‘count’ models (Poisson and Negative Binomial) were considered. Due to the over-dispersion of zero counts, zero-inflated Poisson and Negative Binomial models were also estimated, in which there were two models: a logistic regression looking at the probability of any submission; and a count model conditional on there being a submission.

All estimations were conducted across all 30 imputed datasets with within- and between- variation synthesised using Rubin’s rule. The analysis data consisted of all ward * hour observations for which the WardSonar tool was in systematic use. i.e., excluding the hours after 10 pm and before 8am; and also excluding those systematic time periods for which the tool seemed to be unused or unavailable, for instance Apple Ward after 44 days. Thus the results should be interpreted as conditional on the WardSonar tool being in general use. A sensitivity analysis was run in which no day-time data were excluded. The reduced dataset contained 4,695 ward by hour observations and the fuller dataset contained 6,300 observations.

In addition to the core design matrix, a Fir ward by weekend interaction dummy variable was added to accommodate the noticeable, but not total, drop-off in submissions on Fir ward during weekends. For the fuller dataset, containing periods of systematic zero counts, additional ad-hoc dummy variables were included, indicating whether the data referred to an Elderflower ward weekend (Fri – Sun) and/or a period in which no data submissions were made. These were only included in the zero prediction stage of the zero-inflated models.

Table 2 shows the best fit Zero-Inflated Negative Binomial model. Because of the large proportion of zero counts, the negative binomial distributional assumption is preferred to the Poisson and the two-stage zero-inflated strategy preferred to the standard traditional count models. The table shows the co-efficient values and standard errors for both stages of the model, with logistic model for predicting zero counts having co-efficient prefixed by Zinf for the zero-inflation part of the model. Positive coefficients in the negative binomial part of the model represent increased volume of responses given a response, but as the zero inflation part of the model models probability of a zero count, then positive Zinf coefficients imply a lower count. Thus, the sign of the estimates should be interpreted differently, depending on which part of the model is being considered.

**Table 2:** Zero-Inflated Negative Binomial model for count WardSonar submissions

	Daytime w/o systematic non-use (*n* = 4,695)			Full daytime sample (*n* = 6,300)		
Parameter	Estimate	Std Error	Pr >|t|	Estimate	Std Error	Pr >|t|
Apple ward	1.120	0.329	0.00	1.120	0.329	0.00
Bramble ward	0.341	0.368	0.35	0.341	0.368	0.35
Cherry ward	0.566	0.242	0.02	0.566	0.242	0.02
Damson ward	0.567	0.273	0.04	0.567	0.273	0.04
Elderflower ward	1.172	0.349	0.00	1.172	0.349	0.00
Fir ward	0.911	0.321	0.00	0.911	0.321	0.00
Weekend	−0.223	0.262	0.40	−0.223	0.262	0.40
Weekend ^a^ Fir ward	−5.854	1.453	< .0001	−5.854	1.453	< .0001
Morning	0.484	0.213	0.02	0.484	0.213	0.02
Afternoon	0.333	0.217	0.12	0.333	0.217	0.12
Evening	0.015	0.580	0.98	0.015	0.580	0.98
Any Incident Lag 1 h	0.450	0.285	0.11	0.450	0.285	0.11
Any Incident Lag 2 h	−0.407	0.463	0.38	−0.407	0.463	0.38
Any Incident Lag 3 h	0.234	0.461	0.61	0.234	0.461	0.61
Any Incident Lag 4 h	−0.082	0.495	0.87	−0.082	0.495	0.87
Any Incident Lag 5 h	−0.373	0.527	0.48	−0.373	0.527	0.48
Days Elapsed	−0.002	0.005	0.73	−0.002	0.005	0.73
Zinf^a^ Intercept	1.444	0.281	< .0001	1.444	0.281	< .0001
Zinf Apple ward	1.175	0.348	0.00	1.175	0.348	0.00
Zinf Bramble ward	0.841	0.354	0.02	0.841	0.354	0.02
Zinf Cherry ward	−0.412	0.265	0.12	−0.412	0.265	0.12
Zinf Damson ward	0.328	0.287	0.25	0.328	0.287	0.25
Zinf Elderflower ward	1.058	0.361	0.00	1.058	0.361	0.00
Zinf Fir ward	0.000	0.000		0.000	0.000	
ZInf Weekend	0.397	0.244	0.10	0.397	0.244	0.10
Zinf Weekend ^a^ Fir ward	−4.523	5.463	0.41	−4.523	5.463	0.41
Zinf Weekend^a^Elderflower				17.585	0.235	
Zinf Morning	0.382	0.210	0.07	0.382	0.210	0.07
Zinf Afternoon	0.997	0.211	< .0001	0.997	0.211	< .0001
Zinf Evening	2.472	0.484	< .0001	2.472	0.484	< .0001
Zinf Any Incident Lag 1 h	−0.491	0.317	0.12	−0.491	0.317	0.12
Zinf Any Incident Lag 2 h	−0.339	0.409	0.41	−0.339	0.409	0.41
Zinf Any Incident Lag 3 h	0.086	0.436	0.84	0.086	0.436	0.84
Zinf Any Incident Lag 4 h	−0.026	0.479	0.96	−0.026	0.479	0.96
Zinf Any Incident Lag 5 h	−0.057	0.497	0.91	−0.057	0.497	0.91
Zinf Days Elapsed	0.016	0.004	0.00	0.016	0.004	0.00
Zinf System Not in Use				19.671	0.222	
_Alpha	0.632	0.201	0.00	0.632	0.201	0.00

^*a*^*Zero-Inflated Negative Binomial model*

For the zero-inflation part of the model there was substantial heterogeneity between wards with, all other things being equal, Apple, Bramble and Elderflower all more likely to post zero submissions at any time of the study and Cherry ward least likely to post zero submissions. The Fir fixed effect was omitted to accommodate a constant term. There were also systematic differences across the time of day, with all periods more likely to have zero submissions compared to midday, and a response much less likely to occur during evenings. As indicated by the graphs, the positive and significant coefficient on Zinf days elapsed indicates a higher likelihood of zero submissions over time.

The coefficients for the lagged incident variables in the zero-inflation part of the model were mostly negative but not statistically significant. The 1 h lagged variable was of the largest magnitude and had the predicted negative sign and was the closest to being statistically significant.

In terms of the conditional count second part of the model, significant differences across wards were again observed. However, in this case, wards Apple and Elderflower were more likely to have higher numbers of submissions, if any submissions should occur. Bramble ward, on the other hand, was not only most likely to submit zero responses, but also submitted the lowest volume, even when a submission was made. There were no time effects bar a slightly increased rate of submissions for Morning when at least one submission was made.

The expected coefficient sign for lagged incidents is positive, but only 2 of the 5 lagged variables had a positive coefficient and none were statistically significant. The coefficient for the lag of one hour, as in the zero-inflated regression, was of the hypothesised direction, largest magnitude and the closest to being statistically significant.

The days elapsed variable was not significant for the second part of the model.

The addition of the periods in which there were no submissions with accommodating dummy variables did not change the results. The estimated coefficients on the systematic non-use and Elderflower weekend demonstrated predictable signs of quasi-complete separation.

The variation in response rates across wards observed in the descriptive plots was found to persist in a multivariate statistical analysis. Similarly, there was a trend for submission rates to become lower over time, and for greater numbers to be submitted at Midday. The two-part statistical model looking at WardSonar responses identified that much of the variation in overall number of reports was due to differences in an underlying probability to submit rather than in the volume or clustering of reports when any submission was made.

The estimated coefficients for lagged incidents was a notable finding. Although nothing was statistically significant in the model, the one hour lag was of the expected direction, had the largest magnitude and was closest to being statistically significant. This finding is broadly true across all estimation models estimated (including those not reported). Indeed in the non zero-inflated models, the coefficient attached to incidents lagged by one was always significant and positive, indicating that a response was more likely in the hour following an incident, all other things being equal. It is only when the mechanism of count generation was split into two in the zero-inflated models that significance was lost. Thus, in the exploratory analysis it is concluded that there is weak evidence to suggest that incidents may increase the probability of a response in the following hour, but no evidence to suggest that the effect has a longer duration.

#### Ward atmosphere

This section addresses the type of response for ward atmosphere and atmosphere direction, given that a response has been made. It represents a secondary part of the analysis considering the impact of ward characteristics on likelihood of a response. This regression used all 602 complete submissions and adopted the core design matrix used to estimate volume of responses. Table 3 shows the estimated coefficients.

**Table 3:** Ordered Logistic Regression Models of Ward Atmosphere and Atmosphere Direction

	Ward Atmosphere			Atmosphere Direction		
	Estimate	Std Error	Pr >|t|	Estimate	Std Error	Pr >|t|
Threshold1	−1.44	0.28	< .0001	−0.45	0.30	0.13
Threshold2	0.47	0.28	0.09	2.44	0.32	< .0001
Threshold3	1.86	0.29	< .0001			
Threshold4	3.22	0.33	< .0001			
Apple ward	−0.20	0.31	0.52	−0.39	0.33	0.24
Bramble ward	−0.19	0.35	0.58	−1.32	0.38	0.00
Cherry ward	−0.19	0.24	0.42	−0.68	0.25	0.01
Damson ward	−0.13	0.28	0.65	−0.82	0.30	0.01
Elderflower ward	0.33	0.32	0.29	−0.46	0.35	0.18
Fir ward	0.00			0.00		
Weekend	−0.26	0.23	0.26	−0.38	0.26	0.14
Morning	−0.05	0.20	0.81	−0.26	0.21	0.22
Afternoon	−0.16	0.20	0.42	−0.03	0.22	0.89
Evening	−1.87	0.60	0.00	−0.75	0.61	0.22
Any Incident Lag 1 h	−0.17	0.25	0.50	0.13	0.28	0.65
Any Incident Lag 2 h	−0.71	0.44	0.10	−0.07	0.50	0.88
Any Incident Lag 3 h	−0.09	0.46	0.85	−0.38	0.54	0.48
Any Incident Lag 4 h	−0.25	0.46	0.59	−0.45	0.59	0.44
Any Incident Lag 5 h	−0.28	0.47	0.55	0.44	0.60	0.47
Days Elapsed	0.01	0.00	0.04	0.01	0.00	0.10

In both cases, the response category was treated as ordered with 5 categories for ward atmosphere (Very calm, Calm, Neither calm nor stormy, Stormy, Very stormy) and 3 for atmosphere direction (getting calmer, no change/same, getting stormy). Due to the ordered categorical nature of the data, ordinal logistic regression models were estimated. The ordered regression model also estimated thresholds between these categories within each regression (4 thresholds for the 5 categories in ward atmosphere and 2 thresholds for the 3 categories).

In the atmosphere model, very little was statistically significant. There appeared to be no variation across wards. Lagged incidents appears to have no impact. The negative and significant coefficient attached to evening indicates that stormier atmospheres were more likely to be reported later in the day. The statistically significant coefficient for days elapsed suggest that although the number of submissions declined over time (see previous analysis) they were more likely to report a calmer atmosphere.

For the atmosphere direction, significant differences were found across wards, with Fir ward responses being more proportionately likely to report the atmosphere is getting calmer than other wards, and Bramble ward being more likely to report the atmosphere deteriorating. No other variable was significant.

Overall, in terms of volume and types of display, there is evidence to suggest greater variation in volume of response rather than type of response across wards and time of day.

#### Predictive potential of WardSonar responses and measurement of contagion period

The final set of regression models looks at whether lagged WardSonar responses can help predict the likelihood of future incidents. Such a link would support the argument that measurements of ward atmosphere can be a proactive instrument in reducing ward incidents.

This relationship was explored by looking at the probability of an incident occurring as a function of fixed ward effects, time of day, and the lag of previous incidents (up to 5 h) and had four models of lagged WardSonar responses: mean ward atmosphere; mean ward atmosphere direction; volume of WardSonar submissions and the lowest atmosphere reading. Simple logistic regression was used. Due to the categorical nature of lagged responses and the low volume of some observed values, quasi-complete separation meant that lags of more than one hour could not be estimated in the data.

All non-night observations were used. This included periods when the WardSonar tool appeared not to be in use at all. To accommodate this, an additional category was created for the WardSonar response: ‘WardSonar not in systematic use’. The coefficient attached to this variable was intended to capture whether the periods for which the tool was not used happened to be periods of high incident occurrence. A significant result could suggest underlying tensions which cause incidents and may also affect the use of the tool.

Tables 4 and 5 together summarise the lagged WardSonar responses. All models exclude a constant term to accommodate explicit dummy variables for all wards.

**Table 4:** Regression models of probability of incident in current period as a function of lagged WardSonar responses (see also Table 5)

Parameter	Lagged Mean Ward Sonar Atmosphere			Lagged Mean Ward Sonar Atmosphere Direction		
	Estimate	Std Error	Pr >|t|	Estimate	Std Error	Pr >|t|
Apple Ward	−2.26	0.15	< .0001	−2.26	0.15	< .0001
Bramble Ward	−2.57	0.15	< .0001	−2.57	0.15	< .0001
Cherry Ward	−3.89	0.23	< .0001	−3.89	0.23	< .0001
Damson Ward	−2.42	0.15	< .0001	−2.42	0.15	< .0001
Elderflower Ward	−3.25	0.19	< .0001	−3.25	0.19	< .0001
Fir Ward	−2.84	0.16	< .0001	−2.84	0.16	< .0001
Weekend	0.03	0.11	0.7848	0.03	0.11	0.7898
Morning	0.01	0.16	0.9504	0.01	0.16	0.9466
Afternoon	0.18	0.13	0.1683	0.18	0.13	0.1676
Evening	−0.03	0.16	0.8508	−0.03	0.16	0.8509
Any Incident Lag 1 h	0.44	0.16	0.0073	0.44	0.16	0.0073
Any Incident Lag 2 h	0.23	0.18	0.2164	0.23	0.18	0.2152
Any Incident Lag 3 h	0.48	0.17	0.0061	0.48	0.17	0.0061
Any Incident Lag 4 h	0.40	0.18	0.026	0.40	0.18	0.0266
Any Incident Lag 5 h	0.15	0.22	0.4787	0.15	0.22	0.4843
Sonar not in systematic use	0.17	0.12	0.1737	0.17	0.12	0.1743
Mean Atmosphere Calm lag 1 h	0.31	0.37	0.4121			
Mean Atmosphere Medium lag 1 h	−0.05	0.75	0.9478			
Mean Atmosphere Stormy lag 1 h	0.06	1.07	0.9522			
Getting Calmer Lag 1 h				0.28	0.63	0.6595
No Change Lag 1 h				0.19	0.44	0.6673
Getting Stormier Lag 1 h				0.17	0.76	0.8198

**Table 5:** Regression models of probability of incident in current period as a function of lagged Wardsonar responses. (See also Table 4)

Parameter	Lagged Ward Sonar Submissions			Lagged Worst Ward Sonar Atmosphere		
	Estimate	Std Error	Pr >|t|	Estimate	Std Error	Pr >|t|
Apple Ward	−2.27	0.15	< .0001	−2.26	0.15	< .0001
Bramble Ward	−2.58	0.15	< .0001	−2.57	0.15	< .0001
Cherry Ward	−3.90	0.23	< .0001	−3.89	0.23	< .0001
Damson Ward	−2.42	0.15	< .0001	−2.41	0.15	< .0001
Elderflower Ward	−3.22	0.18	< .0001	−3.24	0.19	< .0001
Fir Ward	−2.84	0.16	< .0002	−2.84	0.16	< .0001
Weekend	0.04	0.11	0.6762	0.03	0.11	0.7869
Morning	0.01	0.16	0.9495	0.01	0.16	0.9755
Afternoon	0.18	0.13	0.1666	0.17	0.13	0.1823
Evening	−0.03	0.16	0.8613	−0.03	0.16	0.8495
Any Incident Lag 1 h	0.44	0.16	0.0073	0.44	0.16	0.0072
Any Incident Lag 2 h	0.22	0.18	0.2399	0.23	0.18	0.2135
Any Incident Lag 3 h	0.48	0.17	0.0054	0.48	0.17	0.0059
Any Incident Lag 4 h	0.39	0.18	0.0296	0.40	0.18	0.0269
Any Incident Lag 5 h	0.16	0.22	0.4665	0.15	0.22	0.5053
Sonar not in systematic use	0.17	0.12	0.1702	0.17	0.12	0.1694
Any Submissions Lag 1 h	0.08	0.06	0.1495			
Any Submissions Lag 2 h	−0.01	0.08	0.9002			
Any Submissions Lag 3 h	0.04	0.07	0.5013			
Any Submissions Lag 4 h	−0.03	0.09	0.6977			
Any Submissions Lag 5 h	−0.01	0.09	0.9195			
Min Atmosphere Very Calm Lag 1 h				0.71	1.05	0.4971
Min Atmosphere Calm Lag 1 h				0.11	0.60	0.8482
Min Atmosphere Neutral Lag 1 h				−0.17	0.71	0.81
Min Atmosphere Stormy Lag 1 h				0.88	0.51	0.0832
Min Atmosphere Very Stormy Lag 1 h				−14.60	20,436	0.9994

A positive (or negative) coefficient on the series of lagged variables indicates increased (reduced) probability of an incident occurring in the current time period.

In all four regressions, the results of the common variables were very consistent. There was significant variation in the rate of incidents between wards. In all models the ‘WardSonar not in use’ category was not statistically significant. This indicates that the periods for which the tool appeared never to have been used were not different from the other periods in terms of numbers of incidents.

In addition, there was no impact of time of day.

Importantly, there was compelling evidence that lagged incidents increase the probability of an incident in the current time period – with statistically significant positive coefficients for 1, 3 and 4 h. The lack of significance for 2 h is puzzling, but overall, the data showed clear evidence of a lingering effect of previous incidents. On the basis of this result we conclude that there is evidence of subsequent contagion and that this lasts for 4 h post-incident.

In all, there was little evidence that lagged WardSonar responses were predictive of future incidents. No category for median atmosphere nor median atmosphere direction showed any statistical power in predicting incidents from the omitted no response category; and nor were the coefficients in the hypothesised directions. For example, the calm category for ward atmosphere has a positive coefficient when a negative coefficient would have been expected. Furthermore, the size of the positive coefficient is greater than that for the stormy category. A value which predicts fewer incidents when calm compared with stormy would have been expected.

There was a positive relationship between the 1 h lag of volume of submissions and an increased rate of incidents, but it was not statistically significant.

However there was weak evidence (significant at 10%) that any reporting of a stormy atmosphere in a period leads to a higher likelihood of an incident in the following hour than if no response. The estimated coefficient for ‘Very stormy’ as the lowest response showed clear signs of quasi-complete separation.

## Discussion

Construction of an hour-by-hour dataset over each ward permitted detailed analysis of the use of the WardSonar tool over the duration of the study and the relationship with recorded incidents. Analysis revealed substantial variation in the volume of reports generated from the tool rather than the levels of ward atmosphere recorded. Tool use was most common in the middle of the day and there was a small but systematic general trend to using the tool less over the duration of the study. There were occasions during which there were no submissions for days or weeks at a time. There were very few (if any) submissions during night-time hours and at weekends.

In terms of the type of responses for both direction and current atmosphere, although a response appeared to be more likely given a recent incident, the type of response was not sensitive to whether there had been an incident or not. In terms of atmosphere, the only seemingly related factor was that evenings lead to greater likelihood of a worse atmosphere being reported, given a submission was made. Although the volume of submissions decreased over time, the probability of a submission reporting a better atmosphere increased slightly. There was surprisingly no variation across wards. In terms of direction of atmosphere, there were significant differences across wards but this was the only significant variable.

Perhaps the most important regressions look at the ability of the tool responses in terms of predicting future incidents and the impact of lagged incidents, which was the de facto measure of contagion. However, this did not generate useful insights regarding the tool’s predictive powers, regardless of which tool output could be used, especially in comparison with the information that a recent lagged incident gives. The most important finding was that analysis did reveal evidence of intertemporal contagion and that this contagion lasted for four hours post incident.

In summary, there were substantial significant differences in the probability of an incident happening across wards, consistent across all models. Similarly, there was strong evidence to suggest that incidences occurring up to 3–4 h prior adversely affected (i.e. increased) the probability of an incident occurring in the current hour. This was consistent across all models and offers a measure of the duration of contagion. There was no evidence to suggest that the medians of responses are useful for predicting the likelihood of an incident in the current period. However there is weak evidence to suggest that if the volume of submissions is high in the last hour then an incident is more likely. Additionally, there is weak evidence to suggest that an incident leads to a greater use of the WardSonar tool in the following hour but does not appear to influence the type of response. The presence of any individual ‘Stormy’ response or an increased volume of submissions in an hour is associated with an increased likelihood of an incident in the next hour, but is not as strong a signal as is given by recent incidents.

### Contagion/predictive potential of WardSonar

In terms of contagion, the hour-by-hour analysis of incident data showed that that the occurrence of one incident leads to increased probability of further incidences in the next four hours. This not only reflects what is reported in the literature [12, 62, 63]and by stakeholders in the current study, but also narrows the suggested time periods for measuring contagion suggested by Beck et al. [13] [12] from one day to four hours.

### Implementation and development considerations

Qualitative analysis highlighted factors that seemed to contribute to the lack of reporting by patients. Use of the WardSonar tool did not become normalised over the study period; for example, the data were rarely collected 3 × daily by staff. Some staff and patients said a longer study or one conducted when the wards were settled for a prolonged period would help to embed use of WardSonar in ward routines. Furthermore, study implementation depended to a great extent on the ward managers, who highlighted additional difficulties communicating about WardSonar to staff.

Further development of the tool could include revising implementation strategies e.g. reviewing the role of staff in bringing the device round to patients, working with staff and patients to improve understanding of both the study rationale and the tool’s functionality, and evaluating cost and effort involved in of using the tool [55, 56]. Further analysis of context such as organisational factors may be valuable, given the potential relevance to ward safety.

### Impact of COVID-19 on the study

The COVID-19 pandemic had a considerable impact on the research. Nationally, a significant amount of research was paused [64]. All wards reported some kind of disruption to normal routines, stemming directly or indirectly from the COVID-19 pandemic.

There are implications for the quantity and content of patient reports. The lower volume of reports may have reduced the power of the statistical analyses to identify statistically significant relationships between responses and outcomes. Additionally, we do not know whether or how the decline in use of the tool over the study period related to the pandemic. Notably, the volume of routinely collected data, which is where we find significant findings, was not affected.

Additionally, what patients reported may have been influenced by the pandemic: for instance, external tensions may have been sufficiently powerful to replace usual ward dynamics, disturbing the usual sequences that build up to events like violent incidents. If the study had not taken place during the pandemic, there may have been different outcomes and a different relationship estimated. We can only speculate on how the extraordinary research context may have impacted results.

Pragmatic adjustments to the research design made it possible to deliver the WardSonar study. The monitoring tool was implemented during a significant wave of infections which undoubtedly influenced our ability to access wards, collect data and undertake implementation activities. The complex stresses affecting health staff during and after the 2020 COVID-19 pandemic [65, 66] should be emphasised. The fact that this study remained feasible is testament to their dedication and commitment to improving patient care.

## Conclusions

As far as can be ascertained by the research team, this is the first project to monitor patient perspectives in real-time in acute mental health wards. It provides an additional proactive approach to safety which is novel and unique. The analysis indicates the Wardsonar tool is potentially measuring contagion and may identify the likelihood of future incidents occurring.

The WardSonar tool can be refined, and tested further in a post COVID-19 context. A separate analysis of seclusion incidents could explore possible contagion affecting how incidents in the seclusion room impact staff and patients on the main ward. The finding that use of the tool trailed off over time on most wards may suggest a need to review implementation processes in future work.

The WardSonar study responded to evidence gaps around the large numbers of reported and unreported safety issues on acute mental health wards, in which data are collected retrospectively; and almost none of the data relate to the patient perspective. The WardSonar monitoring tool has a strong patient perspective, which arguably gives it particular relevance for addressing patient safety. The innovative data analysis strategy employed in this nurse-led study addresses a growing awareness of a need for better reporting of quantitative nursing research.

## Data Availability

The datasets generated and/or analysed during the current study are not publicly available due to potentially sensitive content, but are available from the corresponding author on reasonable request.

## Abbreviations

ISRCTN: International Standard Randomised Controlled Trial Number
NHS: National Health Service
PICU: Psychiatric Intensive Care Unit
SAS: Statistical Analysis System
UK: United Kingdom
Zinf: Zero-Inflated
